# Rheological Changes in Bio-Based Filaments Induced by Extrusion-Based 3D Printing Process

**DOI:** 10.3390/ma17153839

**Published:** 2024-08-02

**Authors:** Antonella Patti, Stefano Acierno

**Affiliations:** 1 Department of Civil Engineering and Architecture (DICAr), University of Catania, Viale Andrea Doria 6, 95125 Catania, Italy; 2 Department of Engineering, University of Sannio, Piazza Roma 21, 82100 Benevento, Italy; stefano.acierno@unisannio.it

**Keywords:** polylactide acid (PLA), natural filler, wood-based composites, printing speed, nozzle temperature, rheological properties, degradation, FDM, FFF, MEX

## Abstract

In this work, the authors investigated the impact of extrusion-based printing process on the structural characteristics of bio-based resins through rheological measurements. Two commercially available filaments made from unfilled and wood-filled polylactide (PLA) polymers were considered. Three-dimensional specimens were prepared by printing these filaments under various operating conditions, i.e., changing the extruder temperature and printing rate, and examined using time sweep tests. Specific cycle rheological testing was conducted on pelletized filaments to simulate temperature changes in the printing process. The rheological characteristics of unprocessed materials, in terms of storage (G′) and loss (G″) moduli, were found to be slightly affected by temperature changes. For a pure polymer, the G′ slope at a low frequency decreased over time, showing that the polymer chains evolved from a higher to a lower molecular weight. For wood-filled materials, the G′ slope rose over the testing time, emphasizing the formation of a percolated network of structured filler within the matrix. On the other side, the rheological parameters of both materials were strongly impacted by the printing extrusion and the related conditions. At lower nozzle temperatures (200 °C), by decreasing the printing speed, the G′ and G″ curves became increasingly different with respect to unprocessed resin; whereas at higher nozzle temperatures (220 °C), the influence of the printing speed was insignificant, and all curves (albeit distant from those of unprocessed matrix) mainly overlapped. Considerations on degradation kinetics of both materials during the printing process were also provided by fitting experimental data of complex viscosity with linear correlation over time.

## 1. Introduction

Bio-based thermoplastics derived from renewable resources (polysaccharides, proteins, lipids) or bacterial synthesis, as well as the conventional synthesis of bio-derived or synthetic monomers, are becoming increasingly important as consumers and industry look for alternatives to fossil fuels due to benefits such as biodegradability, no toxicity, renewability, low density, and good mechanical properties [1].

Poly(lactic acid) or poly(lactide) (PLA) is a biodegradable thermoplastic polyester derived from renewable sources that has sparked widespread attention as a possible replacement for commercial petroleum-based polymers such as polyethylene, polypropylene, and polystyrene [2]. Low melt strength, a slow crystallization rate, poor processability, high brittleness, and low service temperature are common flaws of PLA polymer that limit its application [3].

Extrusion is the main operation for handling and shaping thermoplastics and is central to most industrial technologies, such as injection molding, blow molding, cast extrusion, thermoforming, fiber spinning, and 3D printing [4]. The process involves heating polymer-based systems above their melting or glass transition temperatures and pressing molten materials through holes in dies or molds [5]. It requires imposing increased stresses and strain rates to the material through rotating or translating elements of the processing equipment, such as screws or rolls [5].

To manage and optimize extrusion, four key parameters can be controlled: moisture content in the polymer, processing temperature, residence time, and shear stress [6]. Under the effect of the aforesaid parameters, thermoplastics can undergo different degradation mechanisms (thermal [7], hydrolitic [8], thermo-oxidative [9], thermo-mechanical [10]) that result in the shortening of the polymer chains, a reduction in the molecular weight, and a decrease in melt viscosity [6]. Aldhafeeri et al. [11] investigated the effect of the extruder type, screw configuration, screw speed, and feed rate on the degradation of PLA. The results showed that depending on the kind of extruder, increasing the feed rate increased the melt temperature and shear stress but had no significant effect on PLA deterioration. On the other hand, increasing residence time by lowering the screw speed accelerated polymer breakdown. The influence of processing parameters during melt extrusion, such as the extruder temperature, screw rotation speed, and moisture, on the degradation of poly(L-lactide) (PLLA) has been studied in [7]. The authors concluded that the presence of moisture in materials reduced the molecular weight at lower processing temperatures. Processing at higher temperatures and lower screw speeds resulted in a drastic PLLA thermal degradation. Pop et al. [12] examined the structural changes occurring during the 3D printing of commercially available filaments made from poly(acrylonitrile-co-butadiene-co-styrene) (ABS), PLA, and PLA/polyhydroxyalcanoate (PHA) reinforced with bamboo through thermal and spectroscopical characterization. The printing extrusion of bio-based materials (PLA, PLA/PHB/bamboo) resulted in molecular reorientation and reduced crystallinity, while ABS printing generated crosslinking.

Three-dimensional printing technologies are currently capturing scientific researchers’ interest as efficient and simple methods for producing models, prototypes, commodities, and customized products with complex and advanced geometry that respond to specific and sophisticated requirements [13,14]. The necessity for sustainable development in this technology motivated researchers to look into the potential applications of bio-based polymers and composites derived from plant fibers [13,15].

Rheological measurements are crucial for controlling and optimizing polymer manufacturing efficiency. Controlling flow behavior revealed changes in structural properties (e.g., molecular weight and branching) of polymer chains as well as adjustments in the processing parameters (temperature, shear, and pressure) [16]. The complex viscosity of virgin, extruded, and injected PLA-based samples was reported in [17] at various testing temperatures. The rheological characteristics of virgin and extruded materials showed minimal differences; on the other side, the viscosity of injected samples was much lower than the others (virgin and extruded), indicating a reduction in molecular weight caused by mechanical and thermal deterioration. The thermal degradation of PLA/organo-modified clay nanocomposites during processing via an internal mixer and twin-screw extruder was evaluated by rheological time sweep measurements in [18]. PLA degradation was accelerated by organo-modified clay, with the degree of exfoliation determining the effect. The better the filler distribution, the greater the amount of exfoliation, and the harsher the polymer destruction. Depending on the processing temperature, adding a multi-functional reactive polymer (chain extender) to nanocomposites improved thermal stability, generating branched or crosslinked structures without affecting filler distribution inside the matrix.

In this framework, this study verified the degrading effect of bio-based polymer systems under extrusion-based 3D printing through a rheological investigation. Two commercially available filaments have been processed in a printer apparatus by varying the operating conditions (extruder temperature and printing speed). Rheological parameters of virgin and printed materials were recorded over time in isothermal mode or varying the testing temperature. Changes in the storage (G′) and loss (G″) modulus or complex viscosity (η*) in printed specimens, compared to unprocessed resins, were intended as material microstructural changes due to thermo-mechanical stress acting during the printing process under various operating conditions.

## 2. Materials and Methods

### 2.1. Materials

Two commercially available filaments (nominal diameter of 1.75 mm) were employed as raw materials for this study: a pure biopolymer made of polylactide acid (PLA) and a filled biopolymer composed of wood and PLA polymer (PLA + WOOD), supplied by Eumakers (Barletta, Italy). According to the manufacturer, Natureworks’ Ingeo biopolymer 4032D (density = 1.24 g/cm^3^; melting point = 155–170 °C) was used to realize the filaments.

### 2.2. Extrusion-Based Printing Process

A Zortax M200 (Zortax, Olsztyn, Poland) was used in the experimentation. Different working conditions have been established based on the filaments (filled or unfilled PLA) to allow for continuous melt flow outside the nozzle and regular layer deposition on the heated platform. Several attempts were made to print the PLA and PLA + WOOD: the printing speed was varied from −30% to +30% of the default value (0% = 100 mm/s), resulting in 70 and 130 mm/s, respectively, while the nozzle temperature was changed from 200 °C to 220 °C. Table 1 summarized technological parameters selected for each filament throughout the printing process.

In both cases, the bed temperature (T_b_) was set at 70 °C, i.e., close to the glass transition temperature of the feedstock material, to promote adhesion of the first layer to the heated platform and avoid printed object distortion following removal from the bed. The filaments were dried in an oven at 70 °C for 5 h before being extruded or tested. Disc-shaped specimens of 25 mm in diameter and 2 mm in thickness were produced for the rheological characterization.

### 2.3. Characterization Techniques

**Figure 1:**
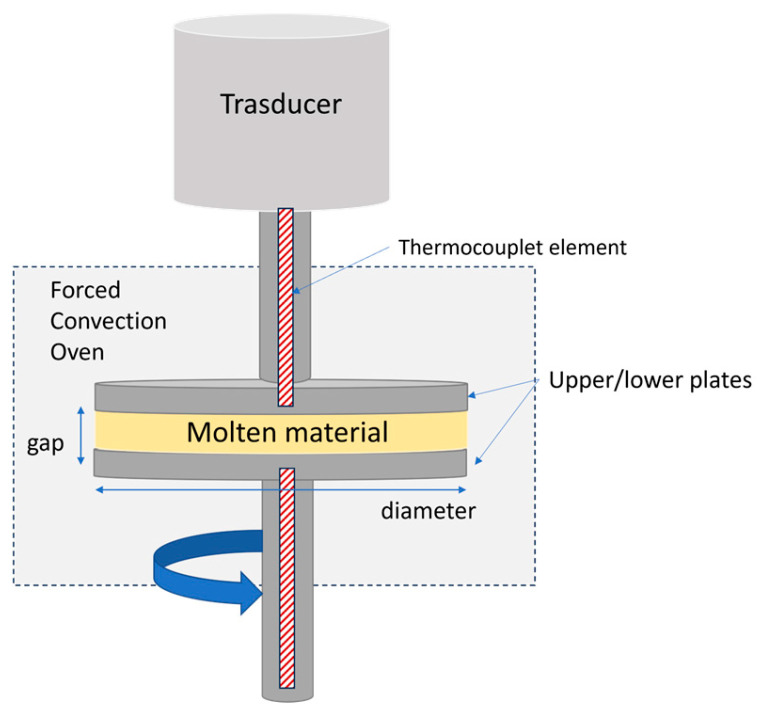
Schematic of strain-controlled rotational rheometer.

The microstructural changes of bio-based polymers under various printing conditions were investigated using rheological measurements on an ARES Rheometer (TA Instruments, New Castle, DE, USA) with 25 mm parallel plates in a nitrogen atmosphere (Figure 1).

During dynamic testing, the material was placed between two heated plates and subjected to a small sinusoidal strain (or stress), with the resulting stress (or strain) being measured.

Under linear conditions, stress (τ, Equation (1)) and strain (γ, Equation (2)) are described by sinusoidal function of time (t):

(1) $$\tau \left(t\right)=\tau_{0}\sin\left(\omega t+\delta \right)$$

(2) $$\gamma \left(t\right)=\gamma_{0}\sin\left(\omega t\right)$$

where ω is the frequency, $\tau_{0}$ is the stress amplitude, $\gamma_{0}$ is the strain amplitude, and δ is the phase shift between the stress signal and the strain signal (δ = 0° corresponds to a perfect elastic, Hookenian, material; δ = 90° to a pure viscous, Newtonian, material).

A complex modulus (G*, Equation (3)) can be defined as follows:

(3) $$G^{*}\left(\omega \right)=\frac{\tau \left(t\right)}{\gamma \left(t\right)}$$

G* is sum of an elastic component, storage modulus (G′, Equation (4)), and a viscous component, loss modulus (G″, Equation (5)):

(4) $$G^{\prime }=\frac{\tau_{0}}{\gamma_{0}}\;\cos\delta$$

(5) $$G^{\prime \prime }=\frac{\tau_{0}}{\gamma_{0}}\;\mathrm{sen}\delta$$

As an alternative to G*, the complex viscosity ($\eta^{*}$, Equation (6)) can be defined as follows:

(6) $$\eta^{*}=\frac{{\left({G\prime }^{2}+G{\prime \prime }^{2}\right)}^{1/2}}{\omega \;}\;\;\;\;\;\;\;\;\;\;$$

Specific experiments were performed to verify the influence of temperature change on the structural properties of the unprocessed filaments. Throughout the 3D printing process, the material is exposed to varying temperatures: it is molten over the melting point, extruded from a nozzle, and deposited as a thin layer on a heated platform, commonly at the glass transition temperatures of extruded materials, where cooling happens.

In this regard, pelletized filaments were subjected to a thermal cycle that consisted of the following steps:

(i) A frequency sweep at 180 °C from 100 to 0.1 rad/s to attest the starting rheological characteristics of two resins;
(ii) A ramp temperature of 20 °C/min up to 200 °C followed by a time sweep for 180 s to simulate extrusion inside the chamber in the molten state;
(iii) A ramp temperature of 20 °C/min up to 100 °C followed by a time sweep for 900 s to simulate the deposition on platform;
(iv) A ramp temperature of 20 °C/min up to 180 °C followed by a time sweep for 180 s to stabilize the conditions;
(v) Five consecutive frequency sweeps at 180 °C from 100 to 0.1 rad/s to assess the material properties following heat treatment.

Dynamic time sweeps were performed on basic pelletized filaments and printed specimens for 1200 s at 180 °C and 1 Hz.

## 3. Results

### 3.1. Printing Attempts

Trials in a different 3D printing setting, i.e., in a different combination of printing speeds (from −30% to +30% of the default value) and nozzle temperature (200 °C, 210 °C, 220 °C) revealed the following:

(i) The maximum allowable speed when the nozzle temperature was 200 °C was equal to +15% (115 mm/s);
(ii) The printing process was always feasible at 210 and 220 °C for each chosen printing speed.

A specific combination of materials’ viscosity and printing speed could lead to a discontinuous flow of molten polymer from the duct, determining a failure in the printing process, according to Equation (7) [19]:

(7) $$v_{print}\eta (T,{\dot{\gamma )}}_{\;}\leq \frac{\Delta P^{max}R^{2}}{2\;L}\left(\frac{n}{3n+1}\right)$$

where $\Delta P^{max}$ is the maximum pressure drop (allowable by printer apparatus) encountered by a non-Newtonian fluid across the conduit of radius *R* and length *L*, $v_{print}$ is the printing speed, $\eta (T,\dot{\gamma )}$ is the material viscosity as a function of the temperature and shear rate (correlated to the printing speed), and n is the shear thinning index.

**Figure 2:**
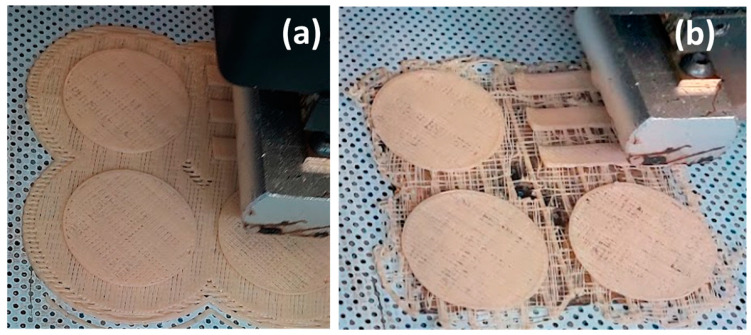
Three-dimensional printed parts by varying the printing speed and nozzle temperature: (**a**) 200 °C and +15%; (**b**) 200 °C and +30%.

At a nozzle temperature of 200 °C (i.e., for a given viscosity and printer apparatus), specific speed values (>15%, 115 mm/s) could result in an exceedance of impressed maximum normal pressure, causing a discontinuous flow of material from the nozzle, incomplete layers, and poor filling in final 3D samples (Figure 2).

### 3.2. Rheological Properties of Basic Materials

**Figure 3:**
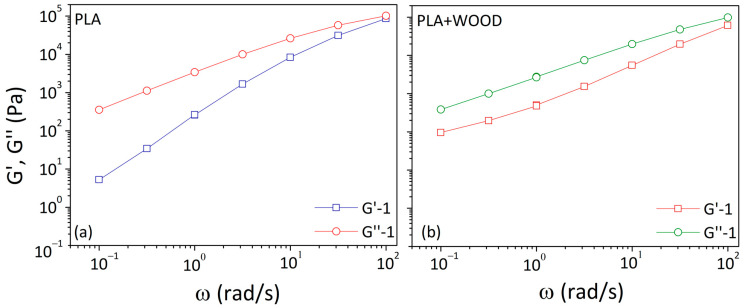
G′ and G″ vs. frequency at 180 °C for: (**a**) PLA and (**b**) PLA + WOOD.

**Figure 4:**
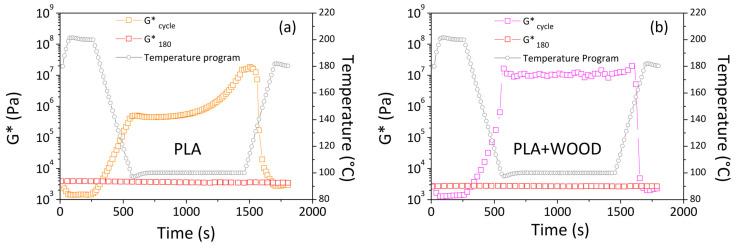
Complex modulus determined at constant temperature of 180 °C (G*_180_) and during temperature program (G*cycle) for: (**a**) PLA and (**b**) PLA + WOOD.

**Figure 5:**
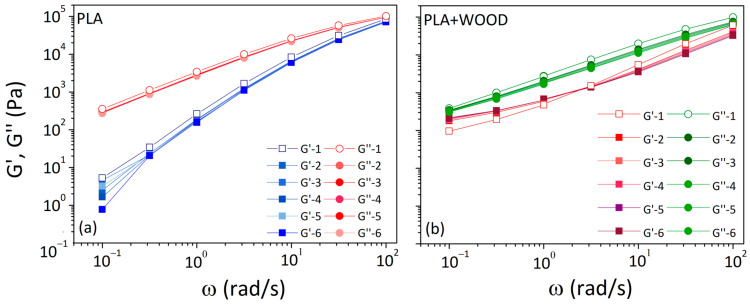
Moduli vs. frequency measured on unprocessed materials (G′-1, G″-1) and during five consecutive tests (G′-6, G″-6) after the temperature program for: (**a**) PLA and (**b**) PLA + WOOD.

Cycle testing on basic pelletized filaments were reported in Figure 3, Figure 4 and Figure 5. Each step for unprocessed materials was detailed below.

The dynamic storage (G′) and loss (G″) moduli against frequency were shown in Figure 3. In both cases, the G′ curves were always lower than the G″ curves, indicating a flow behavior dominated by the viscous component. The G′ and G″ curves were increasingly close at the highest frequency (10^2^ rad/s), even if the crossover frequency was not reached.

In the low frequency range, the G′ curve showed a terminal behavior for PLA (indicating fast segmental relaxation) and a loss of the terminal behavior approaching a plateau for PLA + WOOD (typical of interconnected networks).

Figure 4 shows data collected during the temperature program expressed in terms of the complex modulus (G*).

At 200 °C, G* was approximately 10^3^ Pa for both materials. During cooling at up to 100 °C, the G* increased to 5 × 10^5^ for PLA and more than 10^7^ for PLA + WOOD. At a constant temperature of 100 °C, the G* of PLA increased, reaching values higher than 10^7^ Pa, whereas the G* of PLA + WOOD remained stable and close to 10^7^ Pa.

The difference in response between two materials during this cooling phase was ascribed to different crystallization rates. PLA had a substantially lower crystallization kinetic than PLA + WOOD, and crystallization occurred at a slower rate. This meant that PLA + WOOD crystallized during the cooling ramp temperature, while the PLA crystallized after cooling (during the time sweep).

This assessment found compliance with a preliminary calorimetric investigation [20]. The DSC scan from 200 °C to 20 °C revealed no significant phenomena for the pure polymer during the cooling step and an exothermic peak (characteristic of the crystallization process) with a maximum around 100 °C for the wood-based composite. As previously demonstrated [15,21], the addition of wood filler into PLA polymers tended to considerably reduce the crystallization time because the filler acted as an effective nucleating agent. Extrusion-based 3D products were made with semi-crystalline materials to enhance toughness, wear resistance, stiffness, and strength. On the other hand, the increased crystallization rate was a limiting factor for layer adhesion, preventing macromolecules from interdiffusion across the layer-to-layer interface [16].

At the end of the temperature program, the complex modulus (G*_cycle_, orange points in Figure 4a and pink points in Figure 4b) was lower compared to that measured at a constant temperature of 180 °C (G*_180_, red points).

Figure 5 illustrates data, in terms of G′ and G″, from the first frequency sweep test compared to the five consecutive frequency sweeps following the temperature program.

Small reductions in storage and loss moduli were observed from the first (G′-1, G″-1) to the next five tests (G′-6, G″-6).

In the case of the pure polymer (Figure 5a), the two curves gradually translated downward as the frequency decreased. This could be a result of chain scission and degradation mechanisms that reduced the molecular weight while increasing chain mobility [22]. In the low frequency region (0.1–1 rad/s), all G′ curves presented increasing deviations from G′-1.

In the case of the composite (Figure 5b), at high frequencies (>10 rad/s), the G′ curve after the fifth consecutive frequency sweeps (G′-6) began at lower values than the G′ curve recorded at the beginning (G′-1). Then, G′-6 arrived to equate G′-1 at 3 rad/s and exceeded the G′-1 at low frequencies (<1 rad/s). On the other side, G″-6 began at lower values than G″-1 and rose by decreasing frequency until it equated G″-1. In filled thermoplastic polymers, the interfacial energy between the particles and the matrix was always different. As a result, the particles tended to flocculate under Brownian motion to create a continuous network inside the polymer (i.e., percolation) that hindered molecular motions. Changes in the rheological behavior increased with increasing the filler content. The percolation threshold referred to the minimum filler percentage required to build these networks that drastically changed the rheological behavior from liquid-like to solid-like [23].

In the case of linear polymer melts with a low degree of entanglements, the terminal behavior of G′ and G″ as a function of angular frequency was described by a proportionality law (G′ α ω^2^ and G″ α ω). Table 2 displays the slopes of G′ and G″ in the low frequency region for each test. The slope was calculated by linear fitting points in the range of 0.1–1 rad/s using the Origin program.

For the neat polymer, the G′ slope at low frequencies rose over time from 1.7 to 2.3. A terminal slope of 1.8 was identified as characteristic of a linear polymer with a moderate-molecular-weight distribution of 2–3 [24]. The increase in the G′ slope for PLA was interpreted as polymer chain evolutions from a higher to lower molecular weight. In the composite, the G′ slope gradually approached zero over time. This behavior was attributed to the development of filler/filler and filler/matrix interactions, which produced three-dimensional percolated structures of the particles within the melted polymer.

### 3.3. Rheological Properties of Printed Specimens

#### 3.3.1. Storage Modulus, Loss Modulus, Complex Viscosity

The effect of extrusion-based 3D printing technology on bio-based systems was explored utilizing dynamic time sweeps. The dried filaments were extruded in the printer at various nozzle temperatures and printing rates, realizing 3D printed samples for rheological testing.

**Figure 6:**
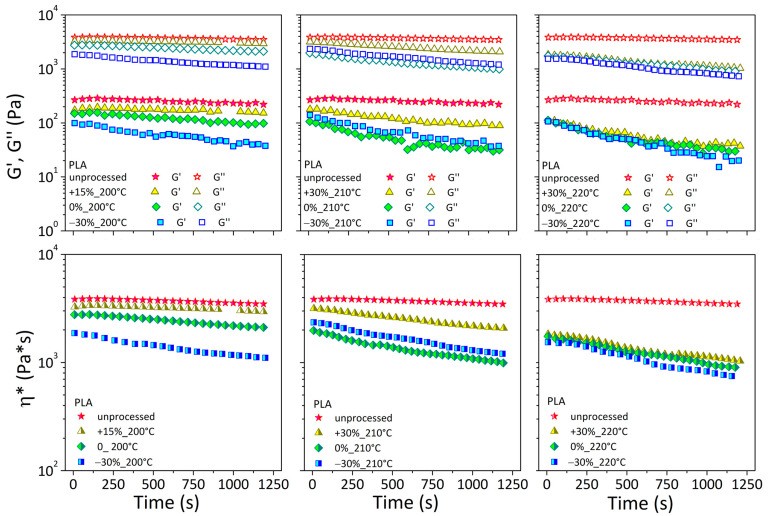
G′, G″, and η* as a function of time for pelletized and printed PLA filaments.

Figure 6 depicts the rheological characteristics as a function of time for unprocessed PLA and related printed specimens under various operating conditions.

Both moduli in the unprocessed polymer (red stars) were stable for up to 1200 s. This result found agreement with rheological measurements by Li et al. [25], for which the PLA material exhibited stable conditions under a nitrogen atmosphere and low temperatures (180 °C).

The rheological signals of printed specimens were always lower than the unprocessed material. At a temperature of 200 °C, G′, G″, and η* remained almost stable when the printing speed was +15% and lowered with decreasing speed. This implied a reduction in thermal stability for printed specimens at low temperatures if the printing speed was reduced. By raising the nozzle temperature, all of the rheological curves of printed specimens get tighter and almost overlapping, with little effect from the printing speed.

**Figure 7:**
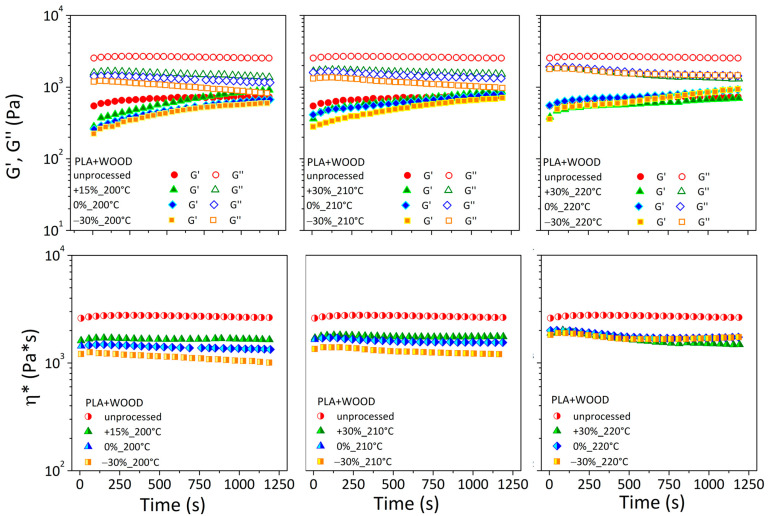
G′, G″, and η* as a function of time for pelletized and printed PLA + WOOD filaments.

Figure 7 depicts the rheological characteristics as a function of time for unprocessed PLA + WOOD and related printed specimens under various extruder temperatures and printing speeds.

The G′ of the unprocessed material rose with time, appearing to reach an asymptotic value, whereas the G″ and η* maintained an almost steady trend.

In the case of printed specimens, the G′ curves started with lower values than the unprocessed material and gradually rose to exceed them; G″ was always lower compared to the unprocessed material and decreased over the testing time, with an effect dependent on printing conditions.

The first outcome (increased G′) was attributed to filler/filler and filler/matrix interactions and the development of three-dimensional filler pathways inside the matrix. This resulted in lower chain mobility and increased melt elasticity. The second event (decreased G″) was linked to the degradation events, material molecular weight reduction, and increased chain mobility. These two effects were recognized as the primary causes that stabilized the complex viscosity of printed composites throughout time.

As the processing temperature increased, in all cases, the differences in printed samples at different speeds diminished.

Strong differences in initial and final values were observed for both neat and filled printed specimens, indicating considerable microstructural changes during testing and as a function of printing conditions (Table 3).

#### 3.3.2. Reduced Complex Viscosity and Material Degradation during the Printing Process

A reduction in molecular weight generated by various degradation mechanisms (thermal, hydrolytic, oxidative, and thermo-mechanical) often causes a decrease in the viscosity of polymer-based systems. Temperature increases induce a rise in intermolecular vibrations and structural alterations in macromolecules. In these conditions, if the chemical bonds are sufficiently stressed, homolytic dissociation may occur, producing fragments with radical end-groups, involving a well-known mechanism called “thermal degradation”. Then, if moisture is present within the polymer structure, both the matrix and water could react, resulting in ester cleavage and the diffusion of low-molecular-weight products out of the polymer as well as in the synthesis of new chain ends. This is a well-known mechanism referred to as “hydrolysis”. During the polymer processing, at high temperatures in an oxygen-depleted atmosphere, the combined effects of temperature and pressure cause the material to degrade. This mechanism is called “thermal-mechanical degradation”. Then, a branching radical chain reaction can be developed in the presence of oxygen through a hydrogen attack or homolytic scission of a carbon–carbon bond. This is the “thermo-oxidative degradation” [6,26].

A large part of the PLA degrading processes includes highly concentrated ester linkages on the main chain. Thermo-hydrolysis, depolymerization, cyclic oligomerization, and intermolecular and intramolecular transesterification are examples of these degradation reactions. Low-molecular-weight species and hydroxyl end groups are considered the primary cause of the decrease in molecular weight at high temperatures, and the thermal degradation was discovered to be caused by random chain scission [27].

Depending on the attack point in the PLA backbone, the end products could be a lactide molecule, an oligomeric ring with more than two repeat units, or acetaldehyde plus carbon monoxide. All of these reactions reduce molecular weight, which results in lower melt viscosity values. Furthermore, the level of mechanical stress applied in the processing can reduce the bonding energy on the backbone of PLA, leading to chain scission [28].

**Figure 8:**
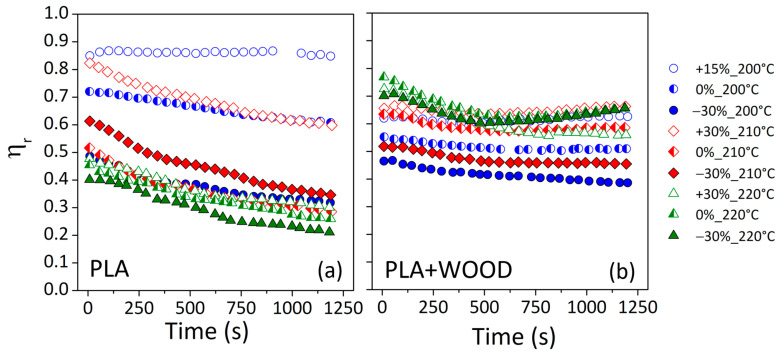
Reduced complex viscosity (*η_r_*) against time for: (**a**) PLA and (**b**) PLA + WOOD.

Figure 8 displays the reduced complex viscosity (*η_r_*) for both materials, i.e., a complex viscosity ratio between the printed specimen ($\eta_{print})$ and unprocessed material ($\eta_{unpr})$ as a function of time (Equation (8)).

(8) $$\eta_{r}\left(t\right)=\frac{\eta_{print}}{\eta_{unpr}}\;\;\;\;\;\;\forall t$$

For the neat polymer (Figure 8a), the *η_r_* curve at the lowest print speed and temperature (−30%_200 °C) (blue solid points) overlapped with curves at 220 °C (green points). Thus, lower extruder temperatures and speeds resulted in polymer breakdown events comparable to higher processing temperatures. Lower extruder temperatures and slower printing speeds resulted in higher viscosities and longer residence times inside the molten chamber. This caused a strong thermo-mechanical stress on polymer chains during the printing process, similar to thermal stress caused by elevated processing temperatures.

Also, for the wood-based composite (Figure 8b), lowest print speed and temperature (−30%_200 °C) related to the worst conditions in term of degradation, for which the highest fall in complex viscosity was detected compared to the unprocessed material (lowest $\eta_{r}$ curve of all). Contrary to the expectation, when the nozzle temperature was of 220 °C, the degrading effects seemed to be less pronounced, and the *η_r_* curves remained superior to all the other conditions. This was attributed to the presence of fillers within polymers and the increased capacity to achieve rheological percolation at high processing temperatures [29].

The percolation reflects a change in the rheological behavior of the composite that leads to a higher degree of system rigidity caused by the filler [29]. At a given filler content, the percolation time was decreased by the increasing temperature [30]. When the processing temperatures were high, the polymer matrix became less viscous, promoting the mobility of the wood particles, which interacted with greater easiness [30,31].

The reduced complex viscosity at zero time (*η_r_*|_t = 0_) was used for assessing material degradation caused by thermo-mechanical stress during extrusion in the printer (Table 4).

Low temperatures and high speed were found to be the best printing parameters for minimizing damage to the neat polymer (*η_r_*|_t = 0_ = 0.85), whereas high nozzle temperatures appeared to be more acceptable for the wood-filled polymer (*η_r_*|_t = 0_ = 0.7).

Thus, the presence of wood fibers in the PLA matrix induced a material stabilization over processing time, slowing down the degradation process and making the final products more durable [32].

#### 3.3.3. Decomposition Kinetics

As reported in the literature [27,33,34], the degradation rate of polymer melts can be described as the rate of chain breaking, the number of chain scissions produced during a known period, according to the following equation (Equation (9)):

(9) $$\frac{1}{\bar{DP}}=\frac{1}{\bar{DP_{0}}}+kt$$

where $\bar{DP}$ and $\bar{DP_{0}}$ are the average polymerization degree at the initial instant and after a time t.

The viscosity of polymers is proportional to the average molar mass. Therefore, the degree of polymerization can be associated with variations in viscosity [27].

For polyester melts (particularly polyethylene terephthalate (PET)), generally, the trend of viscosity as function of time exhibits dual slopes with an initial fast rate and a later slow rate. The first slope is more dependent on moisture absorption by polymers and is due to hydrolysis, whereas the second one is less sensitive to moisture and is due to thermal energy and thermal–oxidative degradation [34]. In the case of dried samples, the first contribution due to the hydrolysis can be considered negligible [34].

When only a single degradation rate takes place, the thermal degradation rate constant for a linear polymer can be calculated using melt viscosities according to Equation (10):

(10) $$\frac{1}{\eta^{\alpha }}=\frac{1}{\eta_{0}^{\alpha }}+kt$$

where η and $\eta_{0}$ are the melt viscosities at the initial instant and after a time *t*, *k* is the thermal degradation rate constant, and α is a constant equal to 1/3.4.

**Figure 9:**
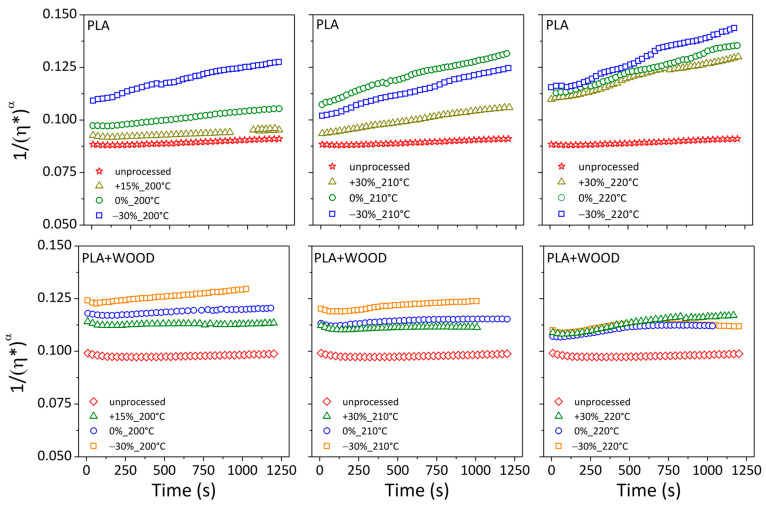
1/η*^α^ vs. time.

Equation (10) was used to fit the inverse of complex viscosity (1/η^α^) vs. time (Figure 9). Fitting parameters are collected in Table 5.

A linear relationship was displayed for the neat polymer in all of the test conditions (R^2^ = 0.97–0.99). Thus, the linear model was regarded to adequately capture deterioration kinetics, and the proposed random main-chain scission appeared to be a true degradation mechanism [35,36]. As expected, the degradation rate constant (*k*) increased by increasing the nozzle temperature and decreasing the printing speed. Printed specimens displayed a degradation kinetic constant (*k*~10^−5^ s^−1^) that was about an order of magnitude higher than the unprocessed material (*k*~10^−6^ s^−1^).

In the case of the composite, the linear model was not always able to match the viscosity data, resulting in a highly varied correlation coefficient (R^2^ = 0.14–0.97). Printing conditions with R^2^ values greater than 0.9 (acceptable) were associated with lower relative viscosity values and a clear monotonous decreasing trend over time (see Figure 8b). In these conditions, degradation processes were the dominant mechanisms influencing the viscosity trend over time. In all other circumstances (R^2^ < 0.9), the reduced viscosity showed a non-monotonous trend in time. This was intended as a sign of other mechanisms, such as the formation of 3D filler structures inside the matrix, which caused an increase in viscosity in contrast to the degradation mechanism.

## 4. Conclusions

This study used rheological measurements to explore changes in the structural properties of bio-based materials by the fused deposition modeling technique. Different considerations were deduced depending on the used filaments (filled or unfilled polylactide acid) and operating printing conditions.

Cycle rheological testing confirmed a slight influence of temperature variations on the rheological behavior of pelletized filaments. During these tests, the G′ slope at low frequencies decreased over time in neat PLA-made specimens, indicating mild polymer degradation and a reduction in molecular weight, whereas in filled-based specimens, the G′ slope at low frequency gradually approached a plateau, indicating the occurrence of a percolated filler network within the polymer matrix.

Time sweep tests proved the stability of virgin pelletized PLA filaments over time as well as the deterioration of printed materials following 3D printing extrusion. The initial values of all rheological parameters in printed specimens were lower than those in unprocessed systems. Then, this decline persisted over time and increased by straining the printing circumstances, such as raising the nozzle temperature and decreasing the printing speed. The combined effect of lower extrusion temperatures (i.e., higher viscosity) and low printing speeds (i.e., longer residence time in the extruder), which resulted mainly in thermo-mechanically induced stress, was found to be comparable to the effect observed when operating at high extrusion temperatures, which resulted mainly in thermally induced stress.

Even in the case of wood-filled polymers, the 3D printing process was found to cause deterioration in the material structure. In these systems, while the polymer decomposed over time (as evidenced by a decrease in G″), the filler formed percolated structures by interacting with the matrix (an increase in G′). These opposing effects were balanced out, resulting in viscosity stability over time (although lower than unprocessed systems).

Kinetic considerations on material degradation allowed us to consider the chain scission model sufficient to describe the degradation mechanism for the pure polymer. In this case, the thermal degradation rate constant of printed specimens was found to be an order of magnitude (~10^−5^ s^−1^) greater than of unprocessed material (10^−6^ s^−1^). In the presence of the wood filler, other mechanisms were assumed to occur (i.e., filler structures in a 3D network) besides chain breakage and scission, hindering the prediction of degrading events through viscosity measurements and linear correlation.

## Figures and Tables

**Table 1:** Process parameters for 3D printing with PLA-based filaments.

	PLA	PLA + WOOD
Nozzle diameter	0.4 mm	0.4 mm
Layer thickness	0.09 mm	0.19 mm
Retraction speed	27 mm/s	20 mm/s
Retraction distance	2.7 mm	1 mm
Infill density	100%	100%
Printing speed	−30% to +30% (70 mm/s to 130 mm/s)	−30% to +30% (70 mm/s to 130 mm/s)
Nozzle temperature	200 °C to 220 °C	200 °C to 220 °C

**Table 2:** G′ and G″ slopes in the low frequency region for PLA and PLA + WOOD.

Test	PLA		PLA + WOOD	
	Slope G′ at ω < 1 rad/s	Slope G″ at ω < 1 rad/s	Slope G′ at ω < 1 rad/s	Slope G″ at ω < 1 rad/s
initial frequency sweep G′-1; G″-1	1.700	0.982	0.693	0.842
1° frequency sweep after thermal program G′-2; G″-2	1.881	0.988	0.518	0.764
2° frequency sweep after thermal program G′-3; G″-3	1.604	0.990	0.514	0.760
3° frequency sweep after thermal program G′-4; G″-4	2.025	0.987	0.497	0.753
4° frequency sweep after thermal program G′-5; G″-5	1.726	0.984	0.485	0.746
5° frequency sweep after thermal program G′-6; G″-6	2.321	0.984	0.465	0.737

**Table 3:** Ratio between initial and final values of G′ and G″ recorded during time sweep. $\frac{{G^{\prime }}_{1200}}{{G^{\prime }}_{0}}$ (or $\frac{{G^{\prime \prime }}_{1200}}{{G^{\prime \prime }}_{0}}$) ≫1 or ≪1 indicates microstructural changes throughtout testing time.

Printing Conditions	PLA		PLA + WOOD	
	$\frac{{G^{\prime }}_{1200}}{{G^{\prime }}_{0}}$	$\frac{{G^{\prime \prime }}_{1200}}{{G^{\prime \prime }}_{0}}$	$\frac{{G^{\prime }}_{1200}}{{G^{\prime }}_{0}}$	$\frac{{G^{\prime \prime }}_{1200}}{{G^{\prime \prime }}_{0}}$
unprocessed	0.80	0.90	1.31	0.99
+15%_200 °C	0.88	0.89	3.22	0.86
0%_200 °C	0.64	0.76	2.59	0.82
−30%_200%	0.37	0.59	2.57	0.73
+30%_210 °C	0.48	0.66	2.23	0.93
0%_210 °C	0.27	0.50	1.96	0.83
−30%_210%	0.30	0.55	2.35	0.78
+30%_220 °C	0.33	0.56	1.80	0.71
0%_220 °C	0.21	0.51	2.58	0.81
−30%_220%	0.19	0.47	2.62	0.81

**Table 4:** Reduced complex viscosity at zero time (η_r_|_t = 0_).

Printing Conditions	*η_r_*|_t=0_	
	PLA	PLA + WOOD
+15%_200 °C	0.85	0.62
0%_200 °C	0.72	0.55
−30%_200 °C	0.48	0.46
+30%_210 °C	0.82	0.65
0%_210 °C	0.51	0.63
−30%_210 °C	0.61	0.52
+30%_220 °C	0.47	0.72
0%_220 °C	0.45	0.77
−30%_220 °C	0.40	0.70

**Table 5:** The intercept $\left(\frac{1}{\eta_{0}^{\alpha }}\right)$, slope (k), and coefficient of determination (R^2^) of the fitting model for PLA and PLA + WOOD.

Printing Conditions	PLA			PLA + WOOD		
	$\frac{1}{\eta_{0}^{\alpha }}$	*k* (s^−1^)	R^2^	$\frac{1}{\eta_{0}^{\alpha }}$	*k* (s^−1^)	R^2^
unprocessed	0.0876	2.76 × 10^−6^	0.974	0.0973	8.30 × 10^−7^	0.338
+15%_200 °C	0.0914	3.32 × 10^−6^	0.926	0.1126	3.86 × 10^−7^	0.139
0%_200 °C	0.0964	7.71 × 10^−6^	0.993	0.1169	3.10 × 10^−6^	0.932
−30%_200 °C	0.1099	1.56 × 10^−5^	0.985	0.1227	6.44 × 10^−6^	0.986
+30%_210 °C	0.0935	1.08 × 10^−5^	0.997	0.1105	1.15 × 10^−6^	0.481
0%_210 °C	0.1091	1.93 × 10^−5^	0.985	0.1125	2.83 × 10^−6^	0.837
−30%_210 °C	0.1021	1.92 × 10^−5^	0.995	0.1187	5.46 × 10^−6^	0.930
+30%_220 °C	0.1098	1.73 × 10^−5^	0.973	0.1082	8.60 × 10^−6^	0.927
0%_220 °C	0.1114	2.04 × 10^−5^	0.995	0.1073	6.13 × 10^−6^	0.847
−30%_220 °C	0.1134	2.64 × 10^−5^	0.987	0.1101	2.69 × 10^−6^	0.445

## Data Availability

The original contriutions presented in the study are included in the article.

## References

[1] Trivedi A.K., Gupta M.K., Singh H. (2023). PLA based biocomposites for sustainable products: A review. Adv. Ind. Eng. Polym. Res..

[2] Simmons H., Kontopoulou M. (2018). Hydrolytic degradation of branched PLA produced by reactive extrusion. Polym. Degrad. Stab..

[3] Nofar M., Sacligil D., Carreau P.J., Kamal M.R., Heuzey M.C. (2019). Poly (lactic acid) blends: Processing, properties and applications. Int. J. Biol. Macromol..

[4] Polychronopoulos N.D., Vlachopoulos J., Mazumder M.A.J., Sheardown H., Al-Ahmed A. (2019). Polymer Processing and Rheology. Functional Polymers.

[5] Capone C., Di Landro L., Inzoli F., Penco M., Sartore L. (2007). Thermal and mechanical degradation during polymer extrusion processing. Polym. Eng. Sci..

[6] Velghe I., Buffel B., Vandeginste V., Thielemans W., Desplentere F. (2023). Review on the Degradation of Poly(lactic acid) during Melt Processing. Polymers.

[7] Taubner V., Shishoo R. (2001). Influence of Processing Parameters on the Degradation of Poly(L-lactide) During Extrusion. J. Appl. Polym. Sci..

[8] Gorrasi G., Pantani R., Di Lorenzo M., Androsch R. (2018). Hydrolysis and Biodegradation of Poly(lactic acid). Synthesis, Structure and Properties of Poly(lactic acid). Advances in Polymer Science.

[9] Srivastava D., Kumar P., Mathur G.N. (2004). Thermo-oxidative degradation studies of ternary blends of polyethylenes. Adv. Polym. Technol..

[10] Pillin I., Montrelay N., Bourmaud A., Grohens Y. (2008). Effect of thermo-mechanical cycles on the physico-chemical properties of poly(lactic acid). Polym. Degrad. Stab..

[11] Aldhafeeri T., Alotaibi M., Barry C.F. (2022). Impact of Melt Processing Conditions on the Degradation of Polylactic Acid. Polymers.

[12] Pop M.A., Croitoru C., Bedő T., Geaman V., Radomir I., Coșnița M., Zaharia S.M., Chicoș L.A., Miloșan I. (2019). Structural changes during 3D printing of bioderived and synthetic thermoplastic materials. J. Appl. Polym. Sci..

[13] Patti A. (2024). Challenges to Improve Extrusion-based Additive Manufacturing Process of Thermoplastics Towards Sustainable Development. Macromol. Rapid Commun..

[14] Morales M.A., Ruiz-Salgado S., Agustín-Serrano R., Zenteno-Mateo B., Rodríguez-Mora J.I. (2023). Design and mathematical modeling of polymeric phases to obtain controlled microporosity materials by 3D printing. Prog. Addit. Manuf..

[15] Liu L., Lin M., Xu Z., Lin M. (2019). Polylactic acid-based wood-plastic 3D printing composite and its properties. BioResources.

[16] Acierno D., Patti A. (2023). Fused Deposition Modelling (FDM) of Thermoplastic-Based Filaments: Process and Rheological Properties—An Overview. Materials.

[17] Pantani R., De Santis F., Sorrentino A., De Maio F., Titomanlio G. (2010). Crystallization kinetics of virgin and processed poly(lactic acid). Polym. Degrad. Stab..

[18] Meng Q., Heuzey M.C., Carreau P.J. (2012). Control of thermal degradation of polylactide/clay nanocomposites during melt processing by chain extension reaction. Polym. Degrad. Stab..

[19] Patti A., Acierno S., Cicala G., Acierno D. (2022). Predicting the Printability of Poly(Lactide) Acid Filaments in Fused Deposition Modeling (FDM) Technology: Rheological Measurements and Experimental Evidence. ChemEngineering.

[20] Patti A., Acierno S., Cicala G., Zarrelli M., Acierno D. (2022). Recovery of Waste Material from Biobags: 3D Printing Process and Thermo-Mechanical Characteristics in Comparison to Virgin and Composite Matrices. Polymers.

[21] Wu W., Wu G., Zhang H. (2017). Effect of wood flour as nucleating agent on the isothermal crystallization of poly(lactic acid). Polym. Adv. Technol..

[22] Techawinyutham L., Siengchin S., Dangtungee R., Parameswaranpillai J. (2019). Influence of accelerated weathering on the thermo-mechanical, antibacterial, and rheological properties of polylactic acid incorporated with porous silica-containing varying amount of capsicum oleoresin. Compos. Part B Eng..

[23] Winter H.H., Mours M. (1997). Rheology of Polymers Near Liquid-Solid Transitions. Adv. Polym. Sci..

[24] Croshaw C., Hamernik L., Ghanbari L., Browning A., Wiggins J. (2022). Melt-state degradation mechanism of poly (ether ketone ketone): The role of branching on crystallization and rheological behavior. Polym. Degrad. Stab..

[25] Lin Z., Guo X., He Z., Liang X., Wang M., Jin G. (2021). Thermal degradation kinetics study of molten polylactide based on Raman spectroscopy. Polym. Eng. Sci..

[26] Vohlidal J. (2021). Polymer degradation: A short review. Chem. Teach. Int..

[27] Maharana T., Mohanty B., Negi Y.S. (2009). Melt-solid polycondensation of lactic acid and its biodegradability. Prog. Polym. Sci..

[28] Cuadri A.A., Martín-Alfonso J.E. (2018). Thermal, thermo-oxidative and thermomechanical degradation of PLA: A comparative study based on rheological, chemical and thermal properties. Polym. Degrad. Stab..

[29] Penu C., Hu G.H., Fernandez A., Marchal P., Choplin L. (2012). Rheological and electrical percolation thresholds of carbon nanotube/polymer nanocomposites. Polym. Eng. Sci..

[30] Zhang C., Wang P., Ma C.A., Wu G., Sumita M. (2006). Temperature and time dependence of conductive network formation: Dynamic percolation and percolation time. Polymer.

[31] Wu G., Asai S., Zhang C., Miura T., Sumita M. (2000). A delay of percolation time in carbon-black-filled conductive polymer composites. J. Appl. Phys..

[32] Patti A., Acierno S., Cicala G., Acierno D. (2024). Aging effects on the viscoelastic behaviour of products by fused deposition modelling (FDM) made from recycled and wood-filled polymer resins. Eur. J. Wood Wood Prod..

[33] Speranza V., De Meo A., Pantani R. (2014). Thermal and hydrolytic degradation kinetics of PLA in the molten state. Polym. Degrad. Stab..

[34] Seo K.S., Cloyd J.D. (1991). Kinetics of hydrolysis and thermal degradation of polyester melts. J. Appl. Polym. Sci..

[35] Daly P.A., Bruce D.A., Melik D.H., Harrison G.M. (2005). Thermal degradation kinetics of poly(3-hydroxybutyrate-co-3-hydroxyhexanoate). J. Appl. Polym. Sci..

[36] Södergård A., Näsman J.H. (1996). Melt stability study of various types of poly(L-lactide). Ind. Eng. Chem. Res..

